# Virulence Comparison of *Salmonella enterica* Subsp. *enterica* Isolates from Chicken and Whole Genome Analysis of the High Virulent Strain *S*. Enteritidis 211

**DOI:** 10.3390/microorganisms9112239

**Published:** 2021-10-28

**Authors:** Luqing Cui, Xiangru Wang, Yue Zhao, Zhong Peng, Pan Gao, Zhengzheng Cao, Jiawei Feng, Fan Zhang, Kaixuan Guo, Min Wu, Huanchun Chen, Menghong Dai

**Affiliations:** 1 The Cooperative Innovation Center for Sustainable Pig Production, Huazhong Agricultural University, Wuhan 430070, China; cuiluqing@webmail.hzau.edu.cn (L.C.); wangxr228@mail.hzau.edu.cn (X.W.); yue1226@webmail.hzau.edu.cn (Y.Z.); pengzhong@mail.hzau.edu.cn (Z.P.); Mrczz180@webmail.hzau.edu.cn (Z.C.); fjw@webmail.hzau.edu.cn (J.F.); zhangxiaofan@webmail.hzau.edu.cn (F.Z.); hzaugkx0612@webmail.hzau.edu.cn (K.G.); chenhch@mail.hzau.edu.cn (H.C.); 2 MOA Key Laboratory of Food Safety Evaluation/National Reference Laboratory of Veterinary Drug Residue (HZAU), Huazhong Agricultural University, Wuhan 430070, China; 3 Department of Biomedical Sciences, School of Medicine and Health Sciences, University of North Dakota, Grand Forks, ND 58203, USA; pan.gao@und.edu (P.G.); min.wu@und.edu (M.W.)

**Keywords:** *Salmonella enterica*, chicken, virulence, whole genome sequencing, *S*. Enteritidis, comparative genomic analysis

## Abstract

Background: *Salmonella* *enterica* is one of the common pathogens in both humans and animals that causes salmonellosis and threatens public health all over the world. Methods and Results: Here we determined the virulence phenotypes of nine *Salmonella* *enterica* subsp. *enterica* (*S. enterica*) isolates in vitro and in vivo, including pathogenicity to chicken, cell infection, biofilm formation and virulence gene expressions. *S*. Enteritidis 211 (SE211) was highly pathogenic with notable virulence features among the nine isolates. The combination of multiple virulence genes contributed to the conferring of the high virulence in SE211. Importantly, many mobile genetic elements (MGEs) were found in the genome sequence of SE211, including a virulence plasmid, genomic islands, and prophage regions. The MGEs and CRISPR-Cas system might function synergistically for gene transfer and immune defense. In addition, the neighbor joining tree and the minimum spanning tree were constructed in this study. Conclusions: This study provided both the virulence phenotypes and genomic features, which might contribute to the understanding of bacterial virulence mechanisms in *Salmonella enterica* subsp. *enterica*. The first completed genomic sequence for the high virulent *S*. Enteritidis isolate SE211 and the comparative genomics and phylogenetic analyses provided a preliminary understanding of *S. enterica* genetics and laid the foundation for further study.

## 1. Introduction

*Salmonella* is a widespread zoonotic pathogen causing food poisoning in humans through infected livestock and poultry, and has caused considerable economic damage worldwide [1]. Analyses indicated that *Salmonella* cases were among the most commonly encountered causes of bacterial foodborne disease globally [2]. *Salmonella* is a highly diverse genus, including two species (*Salmonella bongori* and *Salmonella enterica*), in which *Salmonella enterica* is regarded as the most pathogenic species. In terms of taxonomy, on one side, *Salmonella enterica* can be subdivided into six subspecies such as *Salmonella enterica* subsp. *enterica* (*S. enterica*), *S. salamae*, *S. arizonae*, *S. diarizonae*, *S. houtenae* and *S. indica* [3], and has more than 2600 serovars [4]. On the other hand, *Salmonella* mainly consists of two groups based on the human diseases caused by *Salmonella*: Typhoidal *Salmonella* serotypes that can cause typhoid fever and non-typhoidal *Salmonella* (NTS) serotypes that comprise plenty of serotypes but cannot cause typhoid fever. Typhoidal *Salmonella* is human-restricted, including *Salmonella enterica* subsp. *enterica* serovar Typhi (*S*. Typhi), *S*. Paratyphi and *S*. Sendai [5,6]. NTS mainly refers to *S*. Typhimurium, *S*. Enteritidis, *S*. Dublin, *S*. Anatum, *S*. Indiana, etc. [7,8], and can typically cause a diarrheal disease. It also normally invades sterile sites, leading to bacteremia, meningitis, and other focal infections [9,10]. Also, the invasive non-typhoidal *Salmonella* (iNTS) diseases are often manifested with the characterization of the nonspecific fever similar to malaria and other febrile illnesses, resulting in a higher fatality than the infections by non-invasive strains [9,11]. 

*Salmonella* serotypes may invade multiple host animals differently, can be found in various food sources and bear distinct pathogenic factors, making control of them highly challenging [12]. Clinical pathogenicity of *Salmonella* usually depends on the bacterial load, the infection site of the host, and the production of bacteriotoxin such as ADP-ribosylating toxin protein SpvB [13]. Furthermore, flagella, plasmids, adhesion systems, *Salmonella* pathogenicity islands (SPIs) and its type III secretion systems (T3SS) [14,15] have been demonstrated as the important virulence factors of *Salmonella*. Similar to other enteropathogenic bacteria, *S. enterica* also produces a variety of virulence determinants of adhesion systems, i.e., adhesins, invasins, fimbriae, hemagglutinins, exotoxins and endotoxins [16]. These virulence factors can work alone or in combination to allow *Salmonella* to colonize its host via enhancing the attaching, invading and surviving ability of *Salmonella*. Moreover, these factors help bacteria cells to evade the host’s defense system such as the gastric acidity, gastrointestinal proteases, defensins or aggressins of the intestinal microbiome [2], and can support *Salmonella* initial colonization in the distal small intestine by overcoming the normal resident microbiota [17]. The infections caused by NTS are generally self-limiting and do not proceed beyond the lamina propria. However, some iNTSs have evolved many virulence genes that allow them to invade the intestinal mucosa and proliferate in phagocytes [18,19,20]. Since the widespread use of antibiotic reagents to treat the *Salmonella* infections, antibiotic-resistance of *Salmonella* has been a challenge in recent years [21]. In particular, the resistant strains of *Salmonella* have caused considerable economic losses and even the death of humans in some developing countries [22]. Hence, it is meaningful to investigate the potential virulence mechanisms of *Salmonella* and aggressively tackle its pathogenicity by developing effective treatments.

Although the pathogenic mechanisms of *S*. Typhimurium have been investigated, many other *S. enterica* serovars’ pathogenicity is still unclear. The serovar-specific virulence factors, especially SPIs and virulence plasmids, were involved in the severity of salmonellosis [23]. So far, researchers have primarily used the traditional method, PCR amplification for key virulence genes, to assess the virulence genotype in virulent pathogens [24,25]. However, using this method for high-throughput virulence-related genes screening in the whole genome is limited. Here, we set out to compare the virulence characteristics of nine *S. enterica* isolates recovered from chicken and analyze the genome features of a high-pathogenic isolate *S*. Enteritidis 211 (hereafter also referred to as 211 or SE211) by whole-genome sequencing and genomic comparison to explain its virulence mechanism. Based on genome information and annotated gene functions, this study will provide specific genetic characteristics related to virulence of SE211 and gain insights into potential pathogenic mechanisms in bacterial genome. 

## 2. Materials and Methods

### 2.1. Bacterial Strains and Growth Conditions

Nine *S. enterica* isolates (*S*. Enteritidis 201, *S*. Enteritidis 211, *S*. Typhimurium 206, *S*. Typhimurium 114, *S*. Typhimurium 64, *S*. Typhimurium 62, *S*. Typhimurium 92, *S*. Anatum 76 and *S*. Indiana 94) were recovered from chicken as shown in Table S1, identified and stored at MOA Key Laboratory of Food Safety Evaluation/National Reference Laboratory of Veterinary Drug Residue (HZAU), Huazhong Agricultural University, Wuhan, China [26]. The reference strain *S*. Typhimurium CVCC541, a virulent isolate, was purchased from the Chinese Veterinary Culture Collection (http://cvccinfo.ivdc.org.cn/web/shopcart/ProductsSetting_Virus_Detail.aspx?id=CVCC541, accessed date: 1 August 2018). For in vivo and in vitro studies, overnight bacterial cultures in Luria-Bertani (LB) medium (37 °C, 220 rpm) were subcultured with 100-fold dilution in fresh LB to an OD_600_ of 0.5.

### 2.2. Pathogenicity and Virulence Gene Expression

#### 2.2.1. Acute Infection to Chicken

**Figure 1:**
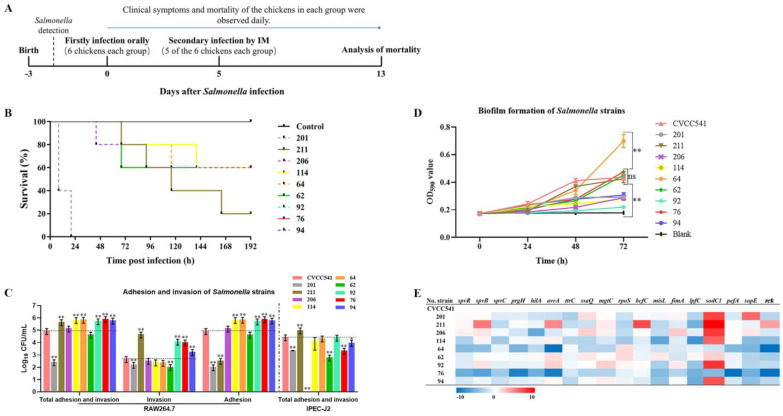
Basic virulence features of the nine *S. enterica* isolates. (**A**,**B**) Acute pathogenicity of the nine *S. enterica* isolates on chickens. (**A**) Design of *Salmonella* acute infection experiment. These newly hatched broiler chickens were firstly detected without *Salmonella* contamination, then orally infected by different *Salmonella* strains in 3 days old, respectively (six chickens each group). Five days post infection, five of the six chickens in each group were secondary infected by intramuscular injection (IM) and observed successively for 8 days. (**B**) Survival curves of these chickens following the infection procedures. The percentages of survival of the chickens infected by each strain were shown. No chicken survived after the infection of *S. enterica* isolate 201, and 20% of the chickens (one chicken) survived after the infection of 211. (**C**) Adhesion and invasion of *S. enterica* bacteria in macrophage (RAW264.7) and IEC (IPEC-J2). The Y-axis is the value of log_10_ CFU/mL of each strain in the cells. (**D**) Biofilm formation of nine *S. enterica* strains at different time points growing in Luria-Bertani (LB) broth at 37 °C. The Y-axis is the OD_590_ value of crystal violet in biofilm. (**E**) The expression of 18 virulence genes in nine *S. enterica* strains isolated from chicken. Horizontal axis represents *S. enterica* strains, one detected gene for one column, red means highly expressed genes, and blue means low expression genes. * *p* ≤ 0.05, ** *p* ≤ 0.01 by one-way analysis of variance (ANOVA) plus Dunnet’s multiple comparison tests compared with CVCC541 in each group respectively; Blank, negative control; Bar, means ± SD; *n* = 3.

A total of 60 newly hatched broiler chickens were purchased from Zaoyang Shigang Agriculture and Animal Husbandry Co. Ltd. (Xiangyang, China), which were not contaminated with *Salmonella* bacteria. These chickens were divided randomly into ten groups of six, including negative control (no infection) and nine experiment groups (201, 211, 206, 114, 64, 62, 92, 76 and 94). The period of the experiment is shown in Figure 1A. Briefly, the 3-day-old chickens were orally inoculated with *Salmonella* bacteria of 2.5 × 10^8^ CFU. Then five infected chickens of each group were secondarily infected by intramuscular injection (IM) with 2.5 × 10^7^ CFU bacteria when they were 8 days old. These chickens were observed continuously for clinical symptoms and mortality.

#### 2.2.2. Adhesion and Invasion Assay

Adhesion and invasion of these *Salmonella* strains (CVCC541, 201, 211, 206, 114, 64, 62, 92, 76 and 94) was determined as previously described [27]. Briefly, 5 × 10^7^ CFU/mL of the *Salmonella* liquids was used to inoculate the monolayers of either macrophage RAW264.7 cells or intestinal epithelial cell (IEC) IPEC-J2 at a multiplicity of infection (MOI) of 100 for three hours. The extracellular unbound bacteria were removed by washing three times of Dulbecco’s Modified Eagle’s Medium (DMEM, Thermo Scientific HyClone, New York, NY, USA) without antibiotics. Then cells were lysed by 0.3% Triton X-100 prepared in Phosphate Buffered Saline (PBS), to determine the total number of adherent and internalized bacteria (Total). To count the invading bacteria (Invasion), the cells need to be incubated in DMEM (gentamicin, 100 mg/mL) for 1 h before lysing. The counts of adherent bacteria (Adhesion) were obtained by the formula: Adhesion = Total − Invasion. Results were averaged from three repeated assays with three technical replicates.

#### 2.2.3. Biofilm Formation Assays

Nine *S. enterica* isolates and a reference strain (CVCC541) were used to detect the production of biofilm which were quantified by crystal violet as described previously [28]. Briefly, overnight bacterial cultures were inoculated with 10-fold dilution to fresh LB in 96-wells polystyrene microtiter plates (Corning, NY, USA). Incubated for 24, 48 or 72 h at 37 °C, the plate wells were treated in the following steps: they were gently washed with PBS, stained using 1% crystal violet (100 μL) for 20 min, gently washed by distilled water, and stained biofilms were resolved in ethanol (100 μL). Finally, liquid absorbance at 590 nm (OD_590_) was measured by an automated microplate reader (TECAN Austria, Grodig, Austria), which represented the biofilm index. The results were averaged by three repeats with six technical replicates each time. Meanwhile, the wells with fresh LB were used as negative control (Blank).

#### 2.2.4. RNA Isolation and Quantitative Reverse Transcription-Polymerase Chain Reaction (RT-qPCR)

Total RNA was prepared from logarithmic phase *S. enterica* cells in LB medium using RNAprep pure Cell/Bacteria Kit (Tiangen biotech Co., Ltd., Beijing, China). cDNA was prepared using HiScript II Q Select RT SuperMix for qPCR (+gDNA wiper) (Vazyme Biotech Co., Ltd., Nanjing, China) and quantified by RT-qPCR (Bio-Rad CFX 96^TM^, Hercules, CA, USA) employing SYBR Green Real time PCR Master Mix (Takara Bio Inc., Kusatsu, Shiga, Japan). Eighteen virulence gene expressions were detected in nine *S. enterica* isolates, including *spvR*, *spvB*, *spvC* (*spv* operon); *prgH*, *hilA*, *avrA* (invasion: SPI-1); *ttrC*, *ssaQ* (proliferation: SPI-2); *mgtC* (magnesium transporter); *rpoS* (virulence regulation); *bcfC*, *misL*, *fimA*, *lpfC* (adherence factors); *sodC1* (stress protein); *pefA* (pilus factor encoded by plasmid), *sopE* (T3SS effector) and *rck* (resistance to complement killing protein). Meanwhile, the ΔΔCt method (2^-ΔΔCt^) was employed to calculate relative gene expressions setting CVCC541 as the control sample. Primers used for RT-qPCR analysis were designed using Primer3 (v.4.1.0), and the nucleotide sequences of primers were contained in Table S2. 

### 2.3. Whole Genome Sequencing and Analysis of SE211

Total genomic DNA of SE211 was extracted using TIANamp Bacteria DNA kit (Tiangen biotech Co., Ltd., Beijing, China). De novo whole-genome sequencing was completed by Shanghai Personalgene Biotechnology. In this project, two libraries (S20K and PE) to different inserts were constructed by the whole genome shotgun (WGS) strategy and sequenced by both next-generation sequencing (NGS) (Illumina HiSeq sequencing platform) and third-generation single-molecule sequencing technology (PacBio Sequel sequencing platform). Then, PacBio sequences were assembled as contigs by the software of HGAP4 [29] and CANU (v.1.6) [30] following as whole genome sequence corrected by NGS sequences using pilon software (v.1.22) [31]. Bacterial sequence type (ST) was determined using multi-locus sequence typing (MLST) analysis [32]. Replicon sequence typing (RST) of plasmid was performed by PlasmidFinder 2.1 and pMLST 2.0 [33,34]. 

The genomic components, encompassing open reading frames (ORFs), tRNA, rRNA, ncRNA, prophage, clustered interspaced short palindromic repeats (CRISPR)-CRISPR-associated (Cas) system and its repeat elements were identified using softwares of GeneMarkS (v.4.32 April 2015) [35] and tRNAscan-SE (v.1.3.1) [36] and some online data bases and tools of Barrnap (0.9-dev) (https://github.com/tseemann/barrnap, accessed date: 29 May 2019), Rfam [37], CRISPR finder [38] and PHASTER (PHAge Search Tool Enhanced Release, http://phaster.ca, accessed date: 29 May 2019) [39]. PathogenFinder 1.1 (https://cge.cbs.dtu.dk/services/PathogenFinder/, accessed date: 20 May 2019) was used in the prediction of a bacteria’s pathogenicity towards human hosts. The virulence factors-related genes in SE211 were predicated by the Database of Virulence Factors of Pathogenic Bacteria (VFDB) [40] using BLAST (blastp 2.6.0+, E-value ≤ 10^–5^, amino acid sequence identity > 60%, coverage of the protein ≥ 70% and the gap sequence < 10 % of the whole length) [41]. KO (KEGG Ortholog) and Pathway for the protein-coding genes were annotated by the KEGG Automatic Annotation Server (KAAS) [42]. The genomic islands were predicted through IslandViewer 4 website [43]. The presence of SPIs (*Salmonella* Pathogenicity Islands) in SE211 was explored by SPIFinder 1.0 (https://cge.cbs.dtu.dk/services/SPIFinder/, accessed date: 25 May 2020) [44]. The genome sequence, gene prediction and non-coding RNA prediction information were integrated into a standard GBK (GenBank) format file, then the circle maps of the chromosome genome and plasmid genome were drawn by CGView [45]. 

The whole genome sequences of SE211 have been deposited in the NCBI under accession numbers CP084532 (chromosome) and CP084533 (plasmid). In addition, eleven publicly available genomes of *Salmonella* were used for comparative genomic analysis with SE211, including four foodborne *S*. Enteritidis strains [EC20121179 (accession no. NC_003197) [46], EC20121175 (accession no. CP007269) [46], P125109 (accession no. NC_011294) [47] and ATCC13076 (accession no. ASM164339v1) [48,49,50]], two zoogenous *S*. Enteritidis strains [EC20130346 (accession no. CP007419) and EC20120051 (accession no. CP007433)] [46], a *S*. Gallinarum strain 287/91 (accession no. AM933173) [47], three *S*. Typhimurium strains [ATCC14028 (accession no. NZ_CP043907) [48,49,50], SL1344 (accession no. FQ312003) [51,52] and LT2 (accession no. NC_003197) [53] and a human pathogen *S*. *arizonae* RKS2983 (accession no. CP006693) [54]]. The chromosomal genome ANI values between SE211 and eleven *Salmonella* genomes were calculated respectively by the website tool (https://www.ezbiocloud.net/tools/ani, accessed date: 20 August 2020) [55], the plasmid sequence ANI between SE211 and the reference strain P125109 were also calculated [P125109 plasmid pSENV (accession no. NZ_CP063701)]. Genomic comparisons were conducted using genome alignment software BLAST Ring Image Generator (BRIG) [56]. Core-genome MLST (cgMLST), neighbor joining tree and minimum spanning tree (MST) were employed by the software of Ridom SeqSphere+ [57], and *Escherichia coli* O157:H7 str. Sakai (accession no. NC_002695) was set as an outgroup in the phylogenetic analysis.

### 2.4. Statistical Analysis

We analyzed in vitro experimental data (such as cell adhesion, invasion, and biofilm formation) using a one-way ANOVA with Dunnet’s multiple comparison tests compared with each group. GraphPad Prism 8 (San Diego, CA, USA) was used for statistical evaluation, asterisks in figures indicated statistical significance: * p ≤ 0.05 and ** p ≤ 0.01.

## 3. Results

### 3.1. Pathogenicity Analysis and Key Virulence Gene Expression

Acute infection on chickens of nine *S. enterica* isolates was performed according to the procedure showed in Figure 1A. After oral infection with 2.5 × 10^8^ CFU of *S. enterica*, four isolates (201, 114, 64 and 206) caused lethality to chicken within 5 days (Table S3), while the chickens infected by the other five isolates (211, 62, 92, 76 and 94) all survived in low spirits or with diarrhea symptoms. Next, we performed the secondary infection to these survived chickens by intramuscular injection. Results showed that the mortality of 201 was 100% in one week, then that of 211 was 80%. In addition, the isolates 206,114, 64 and 62 also had 40% mortality, while 92, 76 and 94 did not show lethality to chickens at the same dose (Table S3, Figure 1B).

Different levels of cell adhesion and invasion were observed in each *S. enterica* strain (Figure 1C). Compared to CVCC541, significant increases of the total adhesion and invasion (Total) to RAW264.7 were observed in isolates 211, 114, 64, 92, 76 and 94, and four of them (211, 92, 76, 94) exhibited remarkably high invasion to RAW264.7. For IEC (IPEC-J2), the Total value of 211 was significantly higher than that of CVCC541, whereas other isolates showed no significant differences or lower adhesion and invasion to IPEC-J2. 

Biofilm formation of the nine isolates was analyzed and shown in Figure 1D, which was positively correlated with the incubation time (from 0 h to 72 h) for each strain. Furthermore, at 72 h, the OD_590_ value of *S. enterica* strain 64 was significantly higher than that of CVCC541, while that of 211, 62 and 76 was not. In sharp contrast, the other five isolates (201, 206, 114, 92 and 94) showed a significantly lower ability of biofilm formation than CVCC541.

The expression (fold change) of eighteen virulence genes of the nine *S. enterica* isolates was detected, in which CVCC541 was set as a control. The log_2_ (fold change) values were taken to produce a heat map (Figure 1E). Apparently, increased expression (red blocks) of these virulence genes in SE211 was observed compared to other strains.

### 3.2. General Features of SE211 Genomes

Based on the studies in vitro and in vivo above, SE211 was preferred to explore potential virulent mechanisms remaining in the genetic level. The genome of SE211 was composed of a chromosome and a virulence plasmid containing two replicons (Table 1). By MLST typing method, SE211 was assigned to ST 11. The genome of SE211 chromosome was 4,679,414 base-pair (bp) length and its plasmid was 59,372 bp in length with 4418 and 86 predicated ORFs, respectively. Their GC content was 52.17% and 51.94%, respectively. In chromosomic genes, approximately 9.1% of the whole genome length (491 genes) were predicted in genomic island areas; and 193 genes of the 4418 ORFs were predicated as virulence factors of pathogenic bacteria. Moreover, eight genes encoded in the plasmid of SE211 were annotated to VFDB genes (Table S4). 

The circle maps of this genome (a chromosome and a plasmid) were displayed in Figure S1. The cluster of orthologous groups (COG) assignment for CDS was represented by the fourth and seventh circles from the inside of the circles and the number of matched genes to each COG category were shown in Figure S1C,D. For the chromosome (Figure S1A,C), 4152 CDS were assigned and classified into 21 of the 26 COG categories, while 46 plasmid genes were annotated into 10 COG categories (Figure S1B,D). rRNA clusters (5s rRNA, 16s rRNA and 23s rRNA) and tRNA were identified on the chromosome of SE211.

### 3.3. Virulence-Related Features in SE211 Genome

According to the online tool of PathogenFinder 1.1, SE211 was predicted to be a human pathogen with the probability of 93.9%. Furthermore, many virulence-associated genes were found in the genome of SE211 (Table S4). Based on the VFDB database, these virulence genes encoded by the chromosome and plasmid were divided into 11 VF classes including fimbrial adherence determinants, non-fimbrial adherence determinants, motility, iron uptake, secretion system and so forth. In detail, each VF class contained several kinds of virulence factors and the related genes as shown in Table S4. In addition, two CRISPR regions were found in the chromosome of SE211, accompanied by eight *cas* genes which belonged to the type I-E CRISPR-Cas system. 

### 3.4. Mobile Genetic Elements

**Figure 2:**
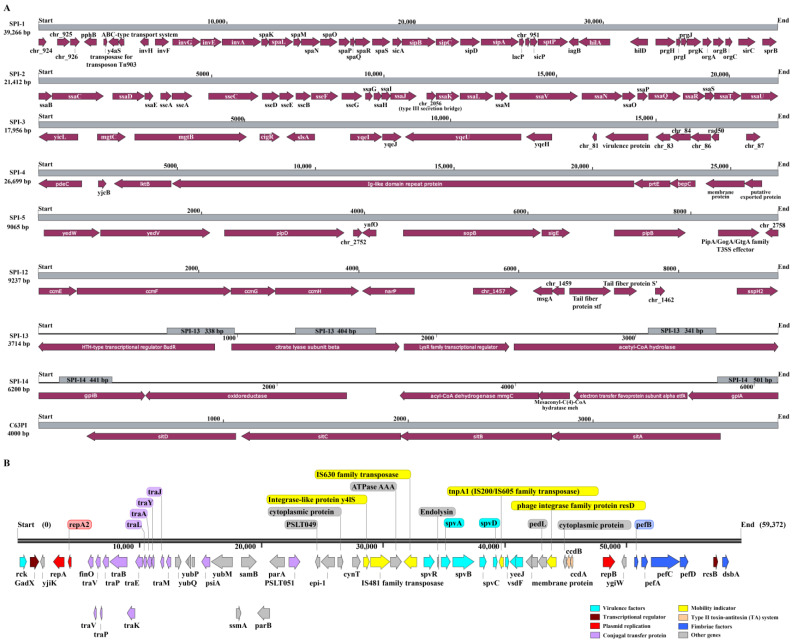
Detailed mapping *Salmonella* pathogenicity islands (SPIs) and plasmid in SE211 genome. (**A**) Schematic representation of the genes carried within the nine SPIs predicated in the genome of SE211. The grey boxes represented the range of SPIs, and the brown boxes showed the CDS encoded in these SPIs regions. (**B**) Gene profile of the virulence plasmid in SE211. Different colors were indicating specific functions as shown at the bottom.

For SE211, 9.1% of the chromosome length were predicted as genomic islands containing 31 GIs (GI1-31, Supplementary Material S1). Among these, GI10 and GI31, GI17-18 and GI21-22 contain T1SS, T3SS, T4SS and T6SS, respectively. Additionally, GI1 and GI13 contain genes of cytochrome c biogenesis, along with GI26, GI5-6, GI-9, GI16, GI21, GI23 and GI30, which are GI-encoding products for fimbrial adhesin and other virulence determinates. Next, we analyzed virulence-related GIs known as SPIs. Results revealed that its genome possessed SPI-1 to SPI-5, SPI-12 to SPI-14, and C63PI, the structure and major functions of these SPIs are shown in Figure 2A and Table S5, respectively. 

Additionally, three incomplete prophages (two in the chromosome and one in the plasmid) and two complete prophages in the chromosome were identified (Table S6), which carried many integrases and transposase genes as shown in Supplementary Material S2. 

For this virulence plasmid, blast analysis indicated that its replicon regions were matched to Incompatibility (Inc) types F (FIB and FIIS, respectively) of replicon sequence type (RST) S1:A-:B22. Genes encoding key virulence factors were harbored in this plasmid, in particular the *Salmonella* plasmid virulence operon (*spvABCD*) and its regulator (*spvR*) (Table S4, Figure 2B). The *spv* plasmid had high homology to the *S*. Enteritidis plasmids belonging to serogroup D that can be found in the NCBI database, for example, the plasmid p1.1-2C7 (accession number: MN125607.1) in *S*. Enteritidis strain isolated from chicken. Additionally, a great number of virulence-related genes were also contained in the plasmid (Figure 2B, in light blue), functioning in complement evasion/serum resistance (*rck*), cell adhesion (*yeeJ*). An unclear functional virulence gene (*vsdF*) was predicated also in this plasmid. Importantly, these virulence genes were flanked by some integrative mobile genetic elements (iMGEs), such as integrase (*y4lS* and *resD*) and transposases (IS481 family, IS630 family and IS200/605 family TnpA1) (Figure 2B, in yellow). Additionally, this plasmid encoded an incomplete conjugal transfer operon (*tra* operon) of 13 genes (*finO* to *traM*) covering 12,577 bp, and some genes (*psiA* and *PSLT051*) encoded conjugation system-related proteins (Figure 2B, in purple). However, the lack of an intact *tra* operon might result in the lack of conjugal transfer for *Salmonella* virulence plasmids. In addition, this *spv* plasmid shares high similarity with the reference virulence plasmid pSENV from *S.* Enteritidis P125109 (Figure S2), the ANI value between two plasmid sequences is 100%. Other genes in this plasmid were closely relevant to fimbrias (*pefABCD*, *dsbA*) (Figure 2B, in blue), transcriptional regulator (*gadX*, *rcsB*) (Figure 2B, in brown), toxin-antitoxin system (Figure 2B, in orange) and so on. 

### 3.4. Comparison of SE211 with Other Salmonella enterica Strains

**Figure 3:**
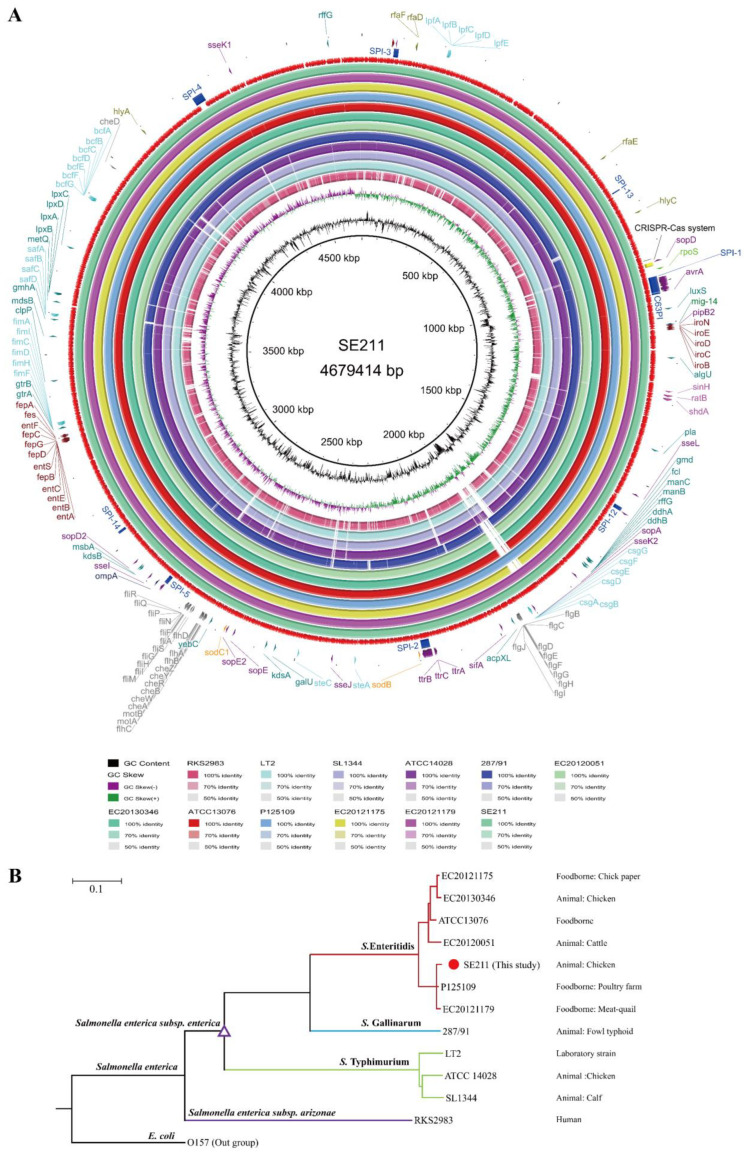
Genome comparisons and hierarchical clustering of twelve *Salmonella enterica* chromosomes. (**A**) Comparison of SE211 with the genomes of EC20121179, EC20121175, P125109, ATCC13076, EC20130346, EC20120051, 287/91, ATCC14028, SL1344, LT2 and RKS2983, displayed as the outer rings inside to outside, respectively. Sequence comparison was performed using BRIG package. DNA identities between different sequences are shown in different colors. The outer two rings show the functional genes encoded in SE211, including the virulence genes, SPI regions (blue) and the position of CRISPR-Cas system (yellow). The outer third ring showed the CDS in the SE211 genome. (**B**) Molecular phylogenetic analysis by neighbor joining method of cgMLST of the 12 *Salmonella enterica* strains. The neighbor joining tree was constructed based on the cgMLST analysis by using the core genomes presents the genomes of the 12 *Salmonella enterica* strains. The tree is drawn to scale, with branch lengths measured in the number of substitutions per site. This analysis involved 12 nucleotide sequences, and evolutionary analyses conducted in Ridom SeqSphere+. *E. coli* O157 was set as the outgroup.

To further assess the pathogenesis, we chose the other 10 *S. enterica* strains and a *S*. *arizonae* strain for comparative genomic analysis against SE211 as shown in Table 2 and Figure 3. Based on the ANI values, SE211 shared most similarities to the four foodborne *S*. Enteritidis strains, followed by two zoogenous *S*. Enteritidis strains, the highly virulent *S*. Gallinarum strain 287/91, three virulent *S*. Typhimurium strains and the human pathogen *S*. *arizonae* RKS2983. High similarity was observed in the genome of the 11 *S. enterica* strains (Figure 3A). Phylogenetic tree construction was based on the neighbor joining analysis of the cgMLST (Figure 3B). It was indicated that these strains were clustered as same as their serotypes. Notably, SE211 is clustered close to the two foodborne strains (P125109 and EC20121179). In particular, P125109 was a human food-poisoning *S*. Enteritidis strain originally isolated in the UK traced back to a poultry farm. The tree also reveals particularly high genetic heterogeneity within the serotypes of *S.* Enteritidis, *S.* Gallinarum and *S.* Typhimurium.

**Figure 4:**
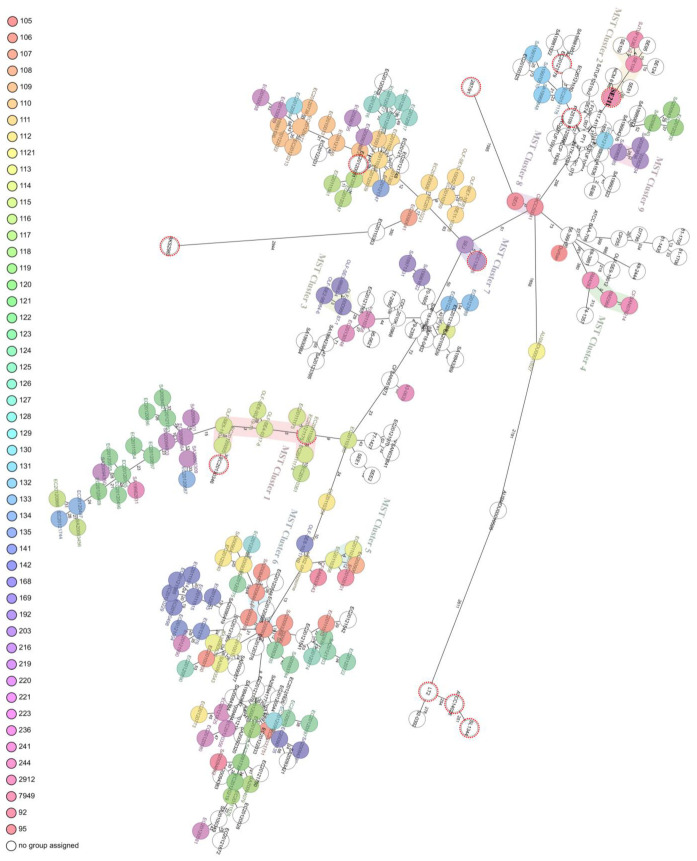
Minimum spanning tree (MST) diagram of 255 *Salmonella enterica* genome samples. In this project included 249 *S.* Enteritidis (three samples with missing values were excluded), a *S.* Gallinarum, three *S.* Typhimurium, a *S. arizonae* and SE211 genome were included. 255 genome samples were represented by these single nodes based on their genotypes and the links between the nodes are based on the distance of the genotypes. The color groups were set according to the cgMLST type. Additionally, there were nine clusters highlighted by different background colors and the name of these clusters were shown as label. The cluster distance threshold is seven alleles difference and at least one other member of the cluster.

The genomes of twelve *Salmonella enterica* strains (eleven *S. enterica* including SE211 and a *S*. *arizonae*) and 246 *S.* Enteritidis genomes downloaded from NCBI were used to perform the cgMLST-based MST diagram (Figure 4). The MST for 258 genome samples was calculated from a Comparison Table (Supplementary Material S3). There are nine MST clusters, and SE211 belonged to the MST Cluster 2. Besides SE211, two *S.* Enteritidis strains isolated from diarrheal patients [SE104 (accession no. NZ_CP050712.1) and SE109 (accession no. NZ_CP050709.1) and a strain from clinic [SJTUF12367v2 (accession no. NZ_CP041176.1) were also included in this cluster. The distances between *S.* Enteritidis with other serotype strains were far.

## 4. Discussion

The virulence features of nine *S. enterica* isolates recovered from chicken were analyzed here. The two *S.* Enteritidis and four of the five *S.* Typhimurium strains had different levels of mortality, while the other three strains were non-fatal. Cell invasion [13] and biofilm [58,59] were considered to be two significant virulence determinants of *Salmonella* pathogenesis and host response depending on virulence factors [4]. For the nine detected *S. enterica*, the virulence genes *spvB*, *avrA*, *bcfC* and *rck* were highly expressed in SE211, which was consistent with its strong virulence phenotypes (80% lethality to chicken, high rate of cell invasion and medium biofilm formation). Otherwise, in *S.* Enteritidis isolate 201 with the highest infection fatality rate (100%), the virulence gene expression was quite different from SE211 but showed similarity to *S.* Typhimurium 206 (40% lethality). Also, other virulence phenotypes were both weak. Moreover, isolates 114, 64 and 62 were all have a lethality of 40%, but showed weak cell invasion in both macrophage and IEC cell models. Interestingly, the three non-lethal isolates (92, 76 and 94) possess higher invasion ability to macrophage but not IEC. The biofilm formation ability of nine isolates were all modest except for the *S*. Typhimurium isolate 64. For these reasons, we thought that the virulence mechanism might be highly activated in SE211 and correlated with its high lethality and cell invasion ability (multiple virulence features), and so it could be a good model strain to elucidate the underlying pathogenic mechanisms. Notably, the virulence genes detected in this study primarily functioned in cell invasion [60,61], bacterial intracellular survival [62] and immune modulation [63]. The prominent expression of the common virulence genes may be important for the increase of bacterial virulence. Altogether, our studies supported that the acute infection assay, the detection of cell invasion, biofilm formation and selected gene expression might be effective ways to determine bacterial virulence levels, which helped the identification of SE211 as a high-virulent *S. enterica* strain. 

Pathogenicity of *Salmonella* was often correlated with virulence factors that can promote colonization and survival of *Salmonella* within hosts [61]. So far, an increasing number of genomes have been sequenced, and the rapid advances in sequencing technology have driven the identification of virulence proteins from genomes. Here, we revealed the whole genome sequences of SE211 and identified its virulence genes according to VFDB. Most of these genes are involved in transmission and infection, such as adhesion, motility, iron uptake, toxin, virulence regulation and secretion. Genes that help *Salmonella* attach to intestinal villi, also can cause interbacterial attachment to facilitate biofilm formation [64,65,66,67]. Besides the chromosome of *S. enterica*, some VFs can also be found in plasmids [68] and the large plasmid probably contributed to bacterial virulence. For example, a 59 kb plasmid in *S*. Enteritidis strains can lead to higher mortality in both mice and chickens than the negative strains [69]. 

Horizontal gene transfer (HGT) was a core event in genome evolution and microbial adaptation to the ecological niche, and genomic islands (GIs) were gene clusters acquired by bacteria in its genome through HGT. GIs were rich in virulence factors (including several SPIs), antibiotic resistance genes and adaptive metabolic pathways, and have high medical and industrial value [70]. We predicted that approximately 9.1% length of genome sequences can be regarded as GIs accompanied with iMGEs (integrase and transposase), supporting a notion that SE211 can actively acquire genomic elements to increase its pathogenicity. Furthermore, some *Salmonella* GIs that are known as unstable pathogenicity islands, possess the ability to cut and transfer between bacteria [71,72]. The excision of GIs was inducible when the *Salmonella* were exposed to macrophage or under a condition of oxidative stress [72]. Five SPIs (SPI-1 to SPI-5) were found to be important for the pathogenesis of *Salmonella* vs. the commensal *E*. *coli* [73], and these SPIs were also existed in the genome of SE211. The *sitABCD* operon (chr_967 to chr_970) in the C63PI genomic region have been shown to encode protein constituents of manganese (II) and iron (II) uptake system which are required for full virulence of *S*. Typhimurium, particularly under oxidative environments of host [74,75].

We showed that two CRISPR loci and eight Cas proteins were found in the genome of SE211. CRISPR-Cas systems broadly existed in prokaryotes for defense against external invasive genetic elements such as plasmid and phage [76,77] and were involved in the regulation of endogenous genes and bacterial virulence [78,79,80]. Bacterial strains possessing CRISPR-Cas systems often had a stronger ability to form biofilm and were more prone to colonize in mouse organs than those lacking CRISPR-Cas systems or core Cas proteins [28,81,82]. In laboratory research, Cas3 of *Pseudomonas aeruginosa* (*P. aeruginosa*) can target *lasR* (bacterial QS regulator) mRNA to dampen the recognition of toll-like receptor 4 (TLR4) and thus diminish the host defense and pro-inflammatory responses in both cell and mouse models [83]. Our recent study conducted in SE211 (*cas3* WT) also supported the assertion that CRISPR-Cas systems could possibly regulate its virulence by impacting the QS system (*lsr* operon) [28]. Using BLAST, we have identified potential candidate genes targeted by CRISPR-Cas system (Supplementary Material S4: CRISPR-Cas system targets predicated by BLAST). Apart from the *lsr* operon genes, some other important genes were also predicated, including a gene in the phage region (chr position_1873096-1873114), a lipoprotein gene (chr_2203) and a fimbriae gene (*fimC*). These genes all contained several base pairs that were continuous concordant with one of the spacers starting from one side. Intriguingly, *cas3* of *P. aeruginosa* inhibited biofilm production through interacting with a chromosomally integrated prophage gene [84,85]. We proposed that the presence of CRISPR-Cas system may confer SE211 the high virulence, such as biofilm formation capacity. 

A prophage is a phage genome which can be inserted and integrated into the bacterial genome, and it plays an important role in both inter-species and inter-strains variability, including virulence gene delivery for the *Salmonella* genus [86,87,88]. Here, we identified five prophage regions in SE211 genome, in which the basic phage proteins (integrase, head, tail, terminase, portal, capsid, plate, transposase) and some bacterial proteins were encoded. Prophages may increase bacterial virulence potential and its survival ability in harsh environments aiming to keep a good condition for the prophage growth [89,90]. Mechanically, the inserted prophage genome could create some new virulence-related features, such as diphtheria [91]. In addition, the prophage may be triggered to produce a virulent phage. Infection of bacteria by such phages might lead to the inter-bacterial transmission of the virulence-related genes, thus increasing the bacteria virulence [92]. It has been reported that *S*. Enteritidis also harbored the CRISPR-Cas systems [93,94], and these lysogenic phages and mobile genetic elements (MGEs) existing in the bacterial genome may encode inhibitory genes for CRISPR-Cas systems that were known as anti-CRISPRs [95,96,97]. Interestingly, since HGT events could contribute to the transfer of virulence factors and development of antibiotic resistance [98], anti-CRISPRs might potentially influence bacterial pathogenesis. 

The genome similarity of SE211 with eleven selected strains is attributed to their same biological characteristics and evolutional environment. Among these strains, EC20121179, EC20121175, P125109 and ATCC13076 were originally isolated from food. Notably, P125109 was highly virulent to human hosts [47] and was closest to SE211 in phylogeny. Additionally, 287/91, ATCC14028, SL1344, LT2 and RKS2983 were either highly virulent to hosts or had wide host ranges including humans [47,53,54]. Collectively, SE211 was confirmed as a high virulent strain as evidenced by its higher pathogenicity in chickens and strong cell adhesion/invasion and biofilm production abilities. Importantly, cgMLST-based MST analysis of 249 *S.* Enteritidis clustered SE211 together with three human isolates. It was a piece of strong evidence for the clonal transfer between chicken and human and the potential pathogenicity of SE211 as a foodborne pathogen to humans.

## 5. Conclusions

This study reported the phenotypic and genetic characteristics of a high virulent *S*. Enteritidis strain SE211. Genotypic analysis through whole-genome sequencing can be exploited to understand pathogenic mechanisms including virulence, antimicrobial resistance, and molecular evolution for outbreak-associated isolates [99]. The genome of SE211 encodes many virulence factors, especially locating in MGEs that contain a virulence plasmid, 31 GIs, and five prophage regions. Importantly, these MGEs were accompanied by iMGEs, such as integrase and transposase. We also identified a complete CRISPR-Cas system in the genome of SE211 that may also help promote its virulence. In addition, the neighbor joining tree and MST showed a clear distance among those genomes. Analysis of genetic and phenotypic features of *S*. Enteritidis may improve our understanding of pathogenic mechanisms and phylogeny, thus developing new strategies for prevention and treatment of *Salmonella enterica* infections. 

## Figures and Tables

**Table 1:** Basic genome information of SE211.

Genomic Contents	Chromosome	Plasmid
Number of ORFs	4418	86
Genome size (bp)	4,679,414	59,372
G + C (%)	52.17	51.94
Genomic islands (number of genes/%)	491/9.1	0
Annotated proteins by Swiss-Prot database	3890	55
Number genes assigned to COG categories	4152	46
Number genes predicted as VFDB	193	8
Number of rRNAs	22	0
Number of tRNAs	80	0
Number of ncRNAs	279	4

ORF, open reading frames; COG, cluster of orthologous groups; VFDB, Database of Virulence Factors of Pathogenic Bacteria.

**Table 2:** Basic features of six *S. enterica* strains and ANI value between these strains with SE211.

Strain	Serotype	Description	ST	cgMLST Type	Chromosome		
					Size	G + C (%)	ANI Value with SE211
SE211	*S*. Enteritidis	Animal: Chicken	11	7949	4,679,414	52.17	-
EC20121179 (CP007272)	*S*. Enteritidis	Foodborne: Meat-quail	11	8746	4,685,848	52.17	99.97%
EC20121175 (CP007269)	*S*. Enteritidis	Foodborne: Chick paper	11	115	4,679,953	52.17	99.97%
P125109 (NC_011294)	*S*. Enteritidis	Foodborne, highly virulent: An outbreak of human food-poisoning in UK traced back to a poultry farm	11	7	4,685,848	52.17	99.97%
ATCC13076 (ASM164339v1)	*S*. Enteritidis	Foodborne	11	216	4,644,776	52.14	99.96%
EC20130346 (CP007419)	*S*. Enteritidis	Animal: Chicken	11	8747	4,685,836	52.18	99.96%
EC20120051 (CP007433)	*S*. Enteritidis	Animal: Cattle	11	222	4,685,846	52.17	99.96%
287/91 (AM933173)	*S*. Gallinarum	Highly virulent: An outbreak of fowl typhoid	331	30	4,658,697	52.20	99.76%
ATCC14028 (NZ_CP043907)	*S*. Typhimurium	Animal: Chicken	19	19	4,870,224	52.2	98.97%
SL1344 (FQ312003)	*S*. Typhimurium	Animal: Calf	19	278	4,878,012	52.18	98.92%
LT2 (NC_003197)	*S*. Typhimurium	Laboratory strain: ATCC700720, virulent	19	2	4,857,450	52.22	98.5%
RKS2983 (CP006693)	*S. arizonae*	Human: Human pathogen	2402	172	4,574,846	51.47	93.32%

ST, Sequence type; cgMLST, Core-genome MLST; ANI, average nucleotide identity.

## Data Availability

The data presented in this study are openly available in NCBI, reference number [CP084532-CP084533].

## Supplementary Materials

The following are available online: https://www.mdpi.com/article/10.3390/microorganisms9112239/s1, Figure S1: Schematic circular genome of SE211 and the cluster orthologous groups (COG) of genes, including a chromosome (A and C) and a plasmid (B and D), Figure S2: Comparison of pSE211 (the plasmid from SE211) with pSENV (the plasmid from P125109), Table S1: Information of nine *S. enterica* isolates, Table S2: Primers used for quantitative reverse transcription-polymerase chain reaction (RT-qPCR) analysis to detect the virulence genotype, Table S3: Oral infection (2.5 × 10^8^ CFU) and secondary infection (2.5 × 10^7^ CFU) of nine *S. enterica* strains to chicken, Table S4: Virulence associated genes in SE211, Table S5: Predicated *Salmonella* pathogenicity islands (SPIs) in SE211, Table S6: The summary of the predicated prophages in the genome of SE211, Supplementary Material S1: GI1-31, Supplementary Material S2: Prophage detail, Supplementary Material S3: MST, Supplementary Material S4: CRISPR-Cas system targets predicated by BLAST.
