# Supplementary material for: Virulence Comparison of Salmonella enterica Subsp. enterica Isolates from Chicken and Whole Genome Analysis of the High Virulent Strain S. Enteritidis 211
Source: Microorganisms. 2021 Oct 28;9(11):2239. doi: 10.3390/microorganisms9112239 (PMC8619400; doi:10.3390/microorganisms9112239)
Supplement: Supplementary file 1 [file microorganisms-09-02239-s001.zip › Figure S1 ans S2.pdf]

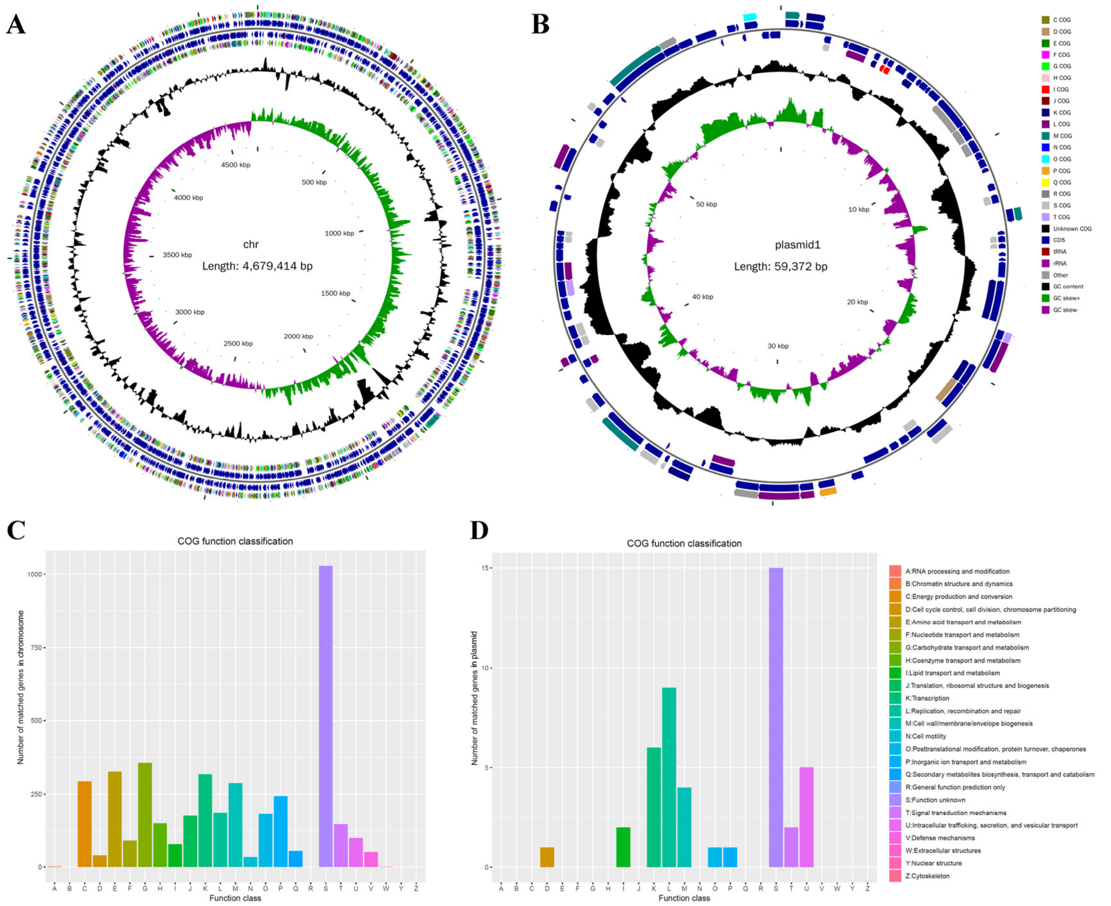

Figure S1. Schematic circular genome of SE211 and the Cluster Orthologous Groups (COG) of genes, including a chromosome (A and C) and a plasmid (B and D). From inside to outside, there were seven rings in both circle maps. The first circle showed the scales, the second circle represented the GC skew, and the third circle meant the GC contents. The fifth and sixth circles showed the position of CDS, tRNA and rRNA in the genome, while the fourth and seventh circles showed the COG categories for the annotated CDS and the number of matched genes to each category (function classification) were shown with the column charts.

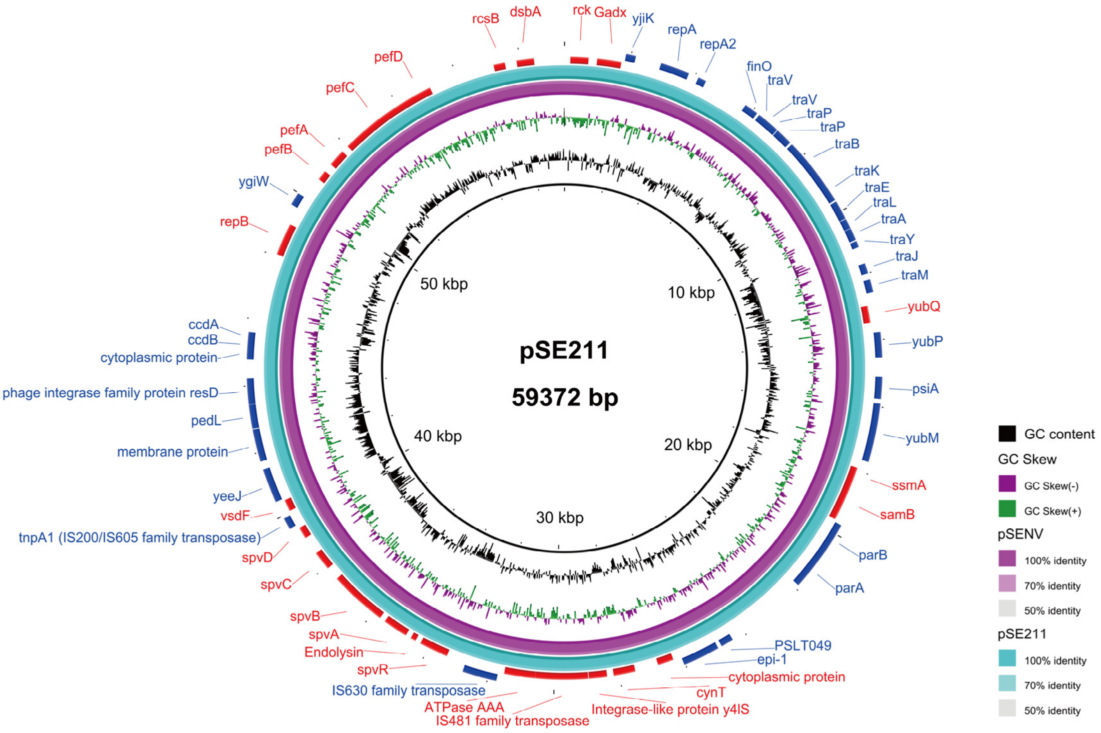

Figure S2. Comparison of pSE211 (the plasmid from SE211) with pSENV (the plasmid from P125109). Sequence comparison was performed using BRIG package. DNA identity between the two sequences were shown in different colors. The outer two rings showed the functional genes existed in pSE211, and the colors showed the gene directions ("red" meant plus strand, "blue" meant minus strand).
