# Supplementary material for: Virulence Comparison of Salmonella enterica Subsp. enterica Isolates from Chicken and Whole Genome Analysis of the High Virulent Strain S. Enteritidis 211
Source: Microorganisms. 2021 Oct 28;9(11):2239. doi: 10.3390/microorganisms9112239 (PMC8619400; doi:10.3390/microorganisms9112239)
Supplement: Supplementary file 1 [file microorganisms-09-02239-s001.zip › Table S1-S6.pdf]

**Table S1.** Information of nine *S. enterica* isolates.

| No. strain | Isolation source | Geographic location | Collection date | Serotypes |
|---|---|---|---|---|
| 62 | Chicken | Henan, China | 2010.10 | *S.* Typhimurium |
| 64 | Chicken | Henan, China | 2010.10 | *S.* Typhimurium |
| 76 | Chicken | Henan, China | 2010.10 | *S*. Anatum |
| 211 | Chicken | Henan, China | 2010.10 | *S.* Enteritidis |
| 114 | Chicken | Hubei, China | 2010.11 | *S.* Typhimurium |
| 92 | Chicken | Hubei, China | 2011.08 | *S.* Typhimurium |
| 94 | Chicken | Hubei, China | 2011.08 | *S.* Indiana |
| 201 | Chicken | Hubei, China | 2011.08 | *S.* Enteritidis |
| 206 | Chicken | Hubei, China | 2011.08 | *S.* Typhimurium |

**Table S2.** Primers used for RT-qPCR analysis to detect the virulence genotype.

| Gene | Primers (5'-3') | Length (bp) |
|---|---|---|
| *16S rRNA* | F: CGGGGAGGAAGGTGTTGTG | 178 |
| | R: GAGCCCGGGGATTTCACATC | |
| *spvR* | F: GCGATGTTACTCATCCCCGT | 125 |
| | R: GGTGTCTCCCGTTTCTTGGT | |
| *spvC* | F: GCAAAGAAACCACTCTGGCG | 156 |
| | R: TGCTTATGATGGGGCGGAAA | |
| *pefA* | F: CCACTGTTTCCTGGGCTTCT | 111 |
| | R: CCGGGTTTTTGGAGTTCACG | |
| *avrA* | F: AGAGTTATGGACGGAAAGAC | 175 |
| | R: AAATACCGCATTCAGAAGAG | |
| *ssaQ* | F: GTCAGGGAGCCCAATGTCAA | 169 |
| | R: CACCTCTGGCGCAAAACATC | |
| *mgtC* | F: AGGCCGCGATCTGTTTACAA | 166 |
| | R: ATGCTCCAGTGAATTGCGGT | |
| *ttrC* | F: TTCGCGGCCGGTATAAATCA | 133 |
| | R: CGGATTTCTCGCTCTGTGGT | |
| *spvB* | F: GATAGCGGGTCACCGTGTAG | 112 |
| | R: GACGGAGAAGTGCTGGTTCA | |
| *sodC1* | F: GATGAACGATGCCCTGTCCA | 170 |
| | R: TACCGTCTTTCATTCCCGGC | |
| *bcfC* | F: CCAGTACGCTGGCGGATAAT | 177 |
| | R: TGTCATCGTCATAGCCGCTC | |
| *rck* | F: AGCTGGCAGTCAGGTAAGCG | 176 |
| | R: TCCTTCACTTCAGCCCTGCC | |
| *fimA* | F: TTTGCTGGCTGTCTCCTCTG | 148 |
| | R: ACGCAGTGTATTGGTGCCTT | |
| *hilA* | F: TTACCCGCTGTATTTATG | 192 |
| | R: TCTCGGATTGAACCTGAT | |
| *lpfC* | F: TGAATCTGACGCAGGCCAAT | 143 |
| | R: CTGAAGGGCACCTTTCTGCC | |
| *misL* | F: TTACTACCGGCGACAGTTCG | 154 |
| | R: GTAGCGTTGTTGCCAAGCTC | |
| *prgH* | F: TGTCGCTGCGCAAAATGAAA | 261 |
| | R: AGCGCTCTCAGCTTTTGACT | |
| *rpoS* | F: ACACCACGCAAGATGACGAT | 147 |
| | R: GACCGATTTCACGGCCTACA | |
| *sopE* | F: TATGCGGGGTCTTTACTCGC | 131 |
| | R: GCAACACACTTTCACCGAGG | |

**Table S3.** Oral infection (2.5 × $10^8$ CFU) and secondary infection (2.5 × $10^7$CFU) of nine *S. enterica* strains to chicken.

| No. strain | Serotypes | Oral infection / Three-day-old chicken | | | Secondary infection by intramuscular injection / Eight-day-old chicken | | |
|---|---|---|---|---|---|---|---|
| | | Total | Number of deaths | Mortality (%) | Total | Number of deaths | Mortality (%) |
| Control | / | 6 | 0 | 0 | 5 | 0 | 0 |
| 201 | *S.* Enteritidis | 6 | 1 | 16.67 | 5 | 5 | 100 |
| 211 | *S.* Enteritidis | 6 | 0 | 0 | 5 | 4 | 80 |
| 206 | *S.* Typhimurium | 6 | 1 | 16.67 | 5 | 2 | 40 |
| 114 | *S.* Typhimurium | 6 | 1 | 16.67 | 5 | 2 | 40 |
| 64 | *S.* Typhimurium | 6 | 1 | 16.67 | 5 | 2 | 40 |
| 62 | *S.* Typhimurium | 6 | 0 | 0 | 5 | 2 | 40 |
| 92 | *S.* Typhimurium | 6 | 0 | 0 | 5 | 0 | 0 |
| 76 | *S.* Anatum | 6 | 0 | 0 | 5 | 0 | 0 |
| 94 | *S.* Indiana | 6 | 0 | 0 | 5 | 0 | 0 |

**Table S4 Virulence associated genes in SE211.**

| VF classes | Virulence factors | Related genes |
|---|---|---|
| Fimbrial adherence determinants | Agf (Thin aggregative fimbriae/curli) | *csgABDEFG, safABCD, steA, steC* |
| | Lpf (Long polar fimbriae) | *lpfABCDE* |
| | Type 1 fimbriae | *fimACDFHI* |
| | Pef (Plasmid-encoded fimbriae) | *pefABCD* |
| | Bcf (Bovine colonization factor) | *bcfABCDEFG* |
| Nonfimbrial adherence determinants | MisL | *misL* |
| | RatB | *ratB* |
| | ShdA | *shdA* |
| | SinH | *sinH* |
| Motility | Flagella | *motA, motB, flgBCDEFGHIJ, flhABCD, fliAFGHIMNPQRS, cheABDRWYZ* |
| Macrophage inducible genes | Mig-14 | *mig14* |
| Regulation | Sigma factor | *rpoS* |
| Iron uptake | Magnesium uptake/transporter | *mgtB, mgtC* |
| | Enterobactin | *entABCEFS, fepABCDG, fes* |
| | Salmochelin | *iroBCDEN* |
| Serum resistance | Rck (Resistance to complement killing) | *rck* |
| | OmpA (Outer membrane protein A) | *ompA* |
| Toxin | Spv (*Salmonella* plasmid virulence) | *spvR, spvB, spvC* |
| | Endotoxin | *rfaDEF* |
| | Hemolysin | *hlyA, hlyC* |
| Stress protein | Superoxide dismutase | *sodB, sodC1* |
| Secretion system | TTSS (SPI-1 encoded) | *hilA, hilD, iacP, iagB, invABCEFGHIJ, orgABC, prgHIJK, sicP, sicA, sipD, spaOPQRS* |
| | TTSS (SPI-2 encoded) | *ssaCDEGHIJKLMNOPQRSTUV, sscA, sscB, sseABCDE, ttrABC* |
| | TTSS-1 translocated effectors | *avrA, sipABC, sopABD, sopE/E2, sptP* |
| | TTSS-2 translocated effectors | *pipB, pipB2, sifA, sopD2, ssaB, sseFGIJL, sseK1/K2, sspH2* |
| Others | *acpXL, algU, clpP, ddhA, ddhB, fcl, galU, gmd, gmhA, gtrA, gtrB, galU, kdsA, kdsB, lpxA, lpxB, lpxC, lpxD, luxS, manB, manC, mdsB, metQ, msbA, pla, rffG, yebC* | |

**Table S5. Predicated *Salmonella* pathogenicity islands (SPIs) in SE211.**

| SPIs | Position | Function |
|---|---|---|
| SPI-1 | 984799…1024064 | Invasion and internalization of bacteria to epithelial cells |
| SPI-2 | 2170656…2192067 | Growth and survival of bacteria within the host cells |
| SPI-3 | 72847…90802 | Adhesion, invasion and intracellular survival |
| SPI-4 | 4237891…4264895 | Adhesion and intracellular survival |
| SPI-5 | 2857400…2866464 | Invasion and intracellular survival |
| SPI-12 | 1574328…1583560 | Intracellular survival and replication |
| SPI-13 | 751826…752163 | Intracellular survival and replication |
| | 752471…752874 | |
| | 754242…754582 | |
| SPI-14 | 3028000…3028440 | Invasion and intracellular survival |
| | 3033521…3034021 | |
| C63PI | 1024961…1028960 | Iron uptake system, sit operon |

©

**Table S6. The summary of the predicated prophages in the genome of SE211.**

| Genome position | ID | Completeness | Number of ORFs | Region (position) | Length (bp) | Specific (gene function) | Number of tRNA |
|---|---|---|---|---|---|---|---|
| Chr | 1 | intact | 62 | 1850594-1913504 | 62911 | integrase, head, tail, terminase, portal, capsid, plate, transposase | 3 |
| | 2 | incomplete | 24 | 2427336-2453127 | 25792 | integrase, lysin | 0 |
| | 3 | intact | 34 | 2662199-2693318 | 31120 | tail, terminase, lysin, integrase, lysis | 0 |
| | 4 | incomplete | 11 | 2897751-2906291 | 8541 | transposase, tail | 0 |
| Plasmid | 1 | incomplete | 9 | 17923-26031 | 8109 | transposase, tail | 0 |
| Total | - | - | 140 | - | - | - | 3 |

Note: “-” represents no more information.
